# Fine Mapping of the Psoriasis Susceptibility Locus *PSORS1* Supports HLA-C as the Susceptibility Gene in the Han Chinese Population

**DOI:** 10.1371/journal.pgen.1000038

**Published:** 2008-03-21

**Authors:** Xing Fan, Sen Yang, Wei Huang, Zhi-Min Wang, Liang-Dan Sun, Yan-Hua Liang, Min Gao, Yue-Qing Ren, Kai-Yue Zhang, Wen-Hui Du, Yu-Jun Shen, Jian-Jun Liu, Xue-Jun Zhang

**Affiliations:** 1 Institute of Dermatology and Department of Dermatology at No. 1 Hospital, Anhui Medical University, Hefei, Anhui, China; 2 The Key Laboratory of Gene Resource Utilization for Genetic Diseases, Ministry of Education, Hefei, Anhui, China; 3 Chinese National Human Genome Center at Shanghai, Shanghai, China; 4 Genome Institute of Singapore, Singapore; The University of Queensland, Australia

## Abstract

*PSORS1* (psoriasis susceptibility gene 1) is a major susceptibility locus for psoriasis. Several fine-mapping studies have highlighted a 300-kb candidate region of *PSORS1* where multiple biologically plausible candidate genes were suggested. The most recent study has indicated *HLA-Cw6* as the primary *PSORS1* risk allele within the candidate region in a Caucasian population. In this study, a family-based association analysis of the *PSORS1* locus was performed by analyzing 10 polymorphic microsatellite markers from the *PSORS1* region as well as *HLA-B*, *HLA-C* and *CDSN* loci in 163 Chinese families of psoriasis. Five marker loci show strong evidence (P<10^−3^), and one marker locus shows weak evidence (P = 0.04) for association. The haplotype cluster analysis showed that all the risk haplotypes are *Cw6* positive and share a 369-kb region of homologous marker alleles which carries all the risk alleles, including *HLA-Cw6* and CDSN*TTC, identified in this study. The recombinant haplotype analysis of the *HLA-Cw6* and CDSN*TTC alleles in 228 Chinese families showed that the *HLA-Cw6* ^−^/CDSN*TTC ^+^ recombinant haplotype is clearly not associated with risk for psoriasis (T∶NT = 29:57, p = 0.0025) in a Chinese population, suggesting that the CDSN*TTC allele itself does not confer risk without the presence of the *HLA-Cw6* allele. The further exclusion analysis of the non-risk *HLA-Cw6* ^−^/CDSN*TTC ^+^ recombinant haplotypes with common recombination breakpoints has allowed us to refine the location of *PSORS1* to a small candidate region. Finally, we performed a conditional linkage analysis and showed that the *HLA-Cw6* is a major risk allele but does not explain the full linkage evidence of the *PSORS1* locus in a Chinese population. By performing a series of family-based association analyses of haplotypes as well as an exclusion analysis of recombinant haplotypes, we were able to refine the *PSORS1* gene to a small critical region where *HLA-C* is a strong candidate to be the *PSORS1* susceptibility gene.

## Author Summary

Psoriasis is a common skin disease with strong genetic risk. The analysis of psoriatic families with multiple affected individuals has identified several genomic regions that are linked (showing linkage evidence) to the development of psoriasis. Of them, the region on 6p21.3 (*PSORS1*) is a well-confirmed major risk locus. The identification of the disease risk gene within the PSOR1 locus, however, has been difficult, largely due to the fact that several genes show evidence for association with the disease development and the evidences are highly correlated and hard to be separated from each other. In this study, we performed a fine mapping study of the *PSORS1* locus in Chinese families with psoriasis. By analyzing recombinant haplotypes that carry different risk-associated genetic variants within different genes, we were able to separate the genetic effects observed within multiple genes and identify strong supporting evidence for *HLA-C* to be the primary risk gene of the *PSORS1* locus. We have further demonstrated that the genetic variation within the *HLA-C* gene does not explain the full linkage evidence at the *PSORS1* locus, suggesting that there might be other risk genes and/or alleles within the region. Our findings have improved our understanding about the genetic complexity of the *PSORS1* locus.

## Introduction

Psoriasis (OMIM*177900) is a common chronic inflammatory skin disorder affecting approximately 2–5% of Caucasian population [1] and 0.123% of the Chinese population [2]. It has long been widely accepted that psoriasis is a complex disease involving multiple genetic and environmental factors. Past genome-wide linkage analyses have identified nine susceptibility loci (designated *PSORS1*–9) and an additional 13 suggestive linkage loci for psoriasis. Among them, the *PSORS1* locus on 6p21.3 is a well-confirmed major susceptibility locus for psoriasis, which accounts for about 30% to 50% of the genetic contribution to the disease [3],[4],[5].

Many fine-mapping studies have been performed to refine the localization of the *PSORS1* gene. By using *HLA* types as markers, Schmitt-Egenolf et al [6],[7] shown that familial psoriasis is associated with the class I end of the EH57.1 haplotype. By performing a linkage disequilibrium (LD) mapping in the Caucasian population, Balendran et al [8] suggested a 285-kb critical region for *PSORS1* between the makers tn821 and *HLA-C*. Oka et al [9] further mapped the *PSORS1* gene to a 111 kb interval telomeric to *HLA-C* through an association analysis in the Japanese population. Similarly, Orru et al's study also highlighted a 70-kb critical region around the *CDSN* gene that is not recombinant with *PSORS1* by identical-by-descent (IBD) haplotyping analysis in a Sardinian population [10]. A 150-kb region telometic to *HLA-C* was also shown to be associated with psoriasis in a Jewish population [11]. More recently, Lench et al [12] performed a SNP-based association analysis and found strong association to a 46-kb interval telomeric to *HLA-C* in both Caucasian and Japanese populations. Helm et al [13] also performed a comprehensive case/control and family-based association study using SNPs and located the *PSORS1* to a haplotype block harboring *HLA-C* and distinct from *CDSN* and *HCR*. Nair et al [14] carried out a comprehensive analysis of the *MHC* region and narrowed the candidate interval for *PSORS1* to a 224-kb region in an American Caucasian population. Taken together, although the results are not totally consistent with each other, these fine-mapping studies generally suggested a critical region of about 300 kb for *PSORS1*, containing *HLA-C* and at least 10 other genes.

As the *PSORS1* locus contains the major histocompatibility complex (*MHC*) that is known to be involved in many autoimmune disorders, *MHC* genes have been intensively evaluated as the candidates for the *PSORS1* susceptibility gene. *HLA-Cw6* has long been known to be associated with susceptibility to psoriasis [15] and was suggested to be a marker allele in LD with the *PSORS1* susceptibility allele because of the lack of its functional role in psoriasis [16],[17],[18]. However, a recent study indicated that there are distinct differential expression patterns of *HLA-C* in psoriasis and eczema, suggesting a functional role of *HLA-C* in psoriasis-related immune response rather than general inflammation [19]. In addition, several other genes within the *PSORS1* locus, including *MICA* [20], *CDSN* [21],[22],[23], *HCR* [24],[25],[26],[27],[28], and *PSORS1C3* [29],[30], were shown to be expressed in skin cells and are, therefore, plausible candidates for the *PSORS1* gene. Other genes like SEEK1 and SPR1 genes were also suggested as the candidates for *PSORS1* [31]. Through a systematic screening of densely distributed SNPs within a 150-kb region around *HLA-C* by family-based analysis, Veal et al [32] identified the strongest association evidence at two SNPs (n. 7 and n. 9) that are located 4 and 7 kb centromeric to *HLA-C*, respectively. Using the haplotype sharing statistic HSS, Foerster et al [33] suggested the localization of *PSORS1* to a small region telomeric of *HLA-C* and indicated that an endogenous retroviral dUTPase should be considered a candidate for the *PSORS1* gene.

Identification of the *PSORS1* susceptibility gene has been difficult due to the existence of extensive LD and multiple biologically plausible candidate genes within the *PSORS1* region [34]. Most of the genes within the 300-kb critical region of *PSORS1* have been shown to be strongly associated with psoriasis [24],[29],[31],[35],[36]. However, the risk alleles of these genes are inseparable from each other due to strong linkage disequilibrium among them. Single marker and haplotype-based association analyses have therefore not been able to determine the primary susceptibility allele. Functional analysis of the known genes within this critical region has also failed to shed light on this problem, because many of these genes have been shown or suggested to be involved in the development of skin tissue and/or the pathogenesis of psoriasis. To overcome this difficulty, Elder and his colleagues genetically dissected the *PSORS1* locus by directly sequencing risk-associated haplotypes and subsequently analyzing recombinant haplotypes that can separate the risk-associated alleles of the several genes [14]. They demonstrated that of the eleven genes within the 300-kb critical region of *PSORS1*, only *HLA-C* and *CDSN* carry nonsynonymous alleles (protein alleles) that are unique to the risk haplotypes of the *PSORS1* locus, making them the more likely candidates to be the *PSORS1* gene. By analyzing the recombinant haplotypes that carry only either the *HLA-C* or *CDSN* risk allele, they further demonstrated that *HLA-C* is more likely to be the *PSORS1* susceptibility gene than the *CDSN* gene.

In this study, we performed the first fine mapping analysis of the *PSORS1* locus in Chinese population. The *PSORS1* locus was confirmed in Chinese population by our previous genome-wide scan analysis [37]. Our previous case-control study had also shown association of certain class I and II *MHC* alleles with psoriasis [38]. However, no fine mapping analyses of *PSORS1* have been done in Chinese population. By performing both population- and family-based association analysis of haplotypes as well as an exclusion analysis of recombinant haplotypes, we were able to refine the *PSORS1* gene to a small critical region where *HLA-C* is a strong candidate of the *PSORS1* susceptibility gene.

## Materials and Methods

### Ascertainment

A total of 228 Han Chinese psoriasis families, including 61 families used in our previous genome-wide linkage study, as well as additional 192 sporadic cases and 192 healthy controls were recruited in this study. All samples were recruited from the Dermatology Department at No. 1 Hospital of the Anhui Medical University by using an ascertainment procedure described previously [37]. The structure and clinical characteristics of all the 228 pedigrees are summarized in Table 1 and Supplementary Table 1. All the samples were recruited with informed content. The study was approved by the Ethics Committee of Anhui Medical University and conducted according to the Declaration of Helsinki Principles.

**Table 1:** Details of Structures of all Pedigrees.

No. of affected members	No. of nuclear families (2 gen)			Generation		Total
	triads	dyads	Sibships	3 gen	4 gen	
1	2	1	0	0	0	3
2	52	33	65	1	0	151
3	17	14	15	13	1	60
≥4	2	0	0	11	1	14
total	73	48	80	25	2	228

Nuclear (2 generation) families are classified as either triads (affected child and two collected parents), dyads (affected child and one collected parent), or Sibships (affected child and one or more collected sibs, with or without parents).

### Genotyping Analysis

10 microsatellite markers (D6S1660, D6S1691, M6S187, C2_4_5, C2_4_4, C1_3_2, C1_2_6, M6S172, D6S273 and D6S1645) were genotyped in 163 families as well as the 192 sporadic cases and 192 healthy controls. The primer information of the 10 microsatellite markers was obtained from the UniSTS Database (http://www.ncbi.nlm.nih.gov/). Marker order and distances were obtained from the National Center for Biotechnology Information (NCBI) database (the build 36.2). HLA-B antigen alleles were also genotyped in the same 163 pedigrees by using DNA-based methods (PCR-SSP) described elsewhere [39].

*HLA-C* and *CDSN* were genotyped in the 163 families and additional 65 affected sib pairs. *HLA-C* alleles were determined by genotyping seven SNPs within *HLA-C* using the method developed by Nair et al [14]. The seven SNPs are located in exons 2 and 3 of the *HLA-C* gene at positions 213, 218, 341, 361, 387, 459, and 540 (NM_002117.4). *HLA-Cw6* genotype can be definitely distinguished from all other alleles by testing the observed genotypes for the seven *HLA-C* SNPs against those produced by all possible combinations of alleles in the IMGT/HLA database [14]. The constructed *HLA-C* haplotypes were retained if they were the only possible outcome given the genotypes of that person and other family members or if the only other choices involved *HLA-C* haplotypes known to be very rare in the study population. The CDSN*TTC allele (defined as CDSN*632T-1249T-1256C) was determined by genotyping three SNPs within *CDSN*. The three polymorphisms within *CDSN* gene are located at positions 632, 1249, and 1256 of the coding sequence (NM_001264).

Genomic DNA was extracted from peripheral blood leukocytes by using standard procedures [40]. Microsatellite markers were genotyped by using a procedure described previously [41]. Briefly, PCR amplifications were performed in a thermocycler 9700TM (Perkin Elmer, Foster City, CA) and PCR products were pooled together and analyzed on an automated ABI Prism 3730 DNA sequencer (Applied Biosystems, Foster City, CA, USA). GeneMapper 4.0 software (Applied Biosystems) was used for determining the fragment sizes of alleles that were further reviewed by two persons independently. All SNPs were genotyped by using the Snap-Shot method of the Applied Biosystems. Pedstats [42] was used to check for non-Mendelian inheritance of alleles and Hardy–Weinberg equilibrium in controls. Unlikely genotypes were further identified by using Merlin (version 1.0.1) [43] and corrected by reviewing the raw genotyping results or re-genotyping if necessary.

### Haplotype Clustering

9-marker haplotypes were constructed in the 163 families by using the genotypes of 7 microsatellite markers as well as the bi-allelic genotypes of *HLA-Cw6* and the quadric-allelic (TTT, TTC, TGC and CTC) genotypes of *CDSN* locus. The haplotype construction was done by using Merlin. Phase ambiguities in the most-likely Merlin haplotypes were then resolved by PHASE (version 2.1.1) whenever the confidence of the phase call was at least 99%. The 9-marker founder haplotypes from the 163 families were then clustered by using an average-distance agglomerative hierarchical method [18] and SPSS 11.5. For haplotypes to be clustered, more than 90% alleles of the haplotypes were required to be typed and of known phase. The criteria for assigning haplotypes to a cluster are ≥80% homogeneity of marker alleles and a minimum number of five founders. Rare haplotypes that are different from a haplotype cluster by only a single repeat (addition or deletion) were merged into the haplotype cluster. The haplotypes that can not be clustered using the above criteria were lumped into a single cluster. The genotypes for *HLA-B* antigen alleles were assigned to the haplotype clusters by the inspection of informative pedigrees.

### Association Test

Single-marker case-control association analysis was performed using the two-tailed chi-squared statistic to compare allele frequency differences. Correction for multiple testing was done by calculating the false discovery rate (FDR) using Benjamini and Hochberg's method [44]. The odds ratio (OR) was calculated by 2×2 contingency tables.

A family-based transmission/disequilibrium test (TDT) was performed by using either single locus genotypes or the haplotype clusters. For all the pedigrees with at least one heterozygous parent where allele or haplotype transmission could be determined, a single affected child was chosen randomly for performing the TDT test. Inferred genotypes and haplotypes of founder individuals without DNA samples were not used in the TDT test due to the potential bias [45].

Family-based conditional association analysis was performed by using the WHAP program version 2.09 [46]. Single trio was randomly selected from each of the 163 families. The 7 microsatellites were treated as multi-allelic locus by using the –usat command. We first evaluated the association of every marker with disease phenotype after controlling for the genotypes of the surrounding loci and then the omnibus association evidence within the region after dropping the *HLA-Cw6* or CDSN*TTC risk allele from the null model.

### Recombination Breakpoints Mapping and Exclusion Analysis

The haplotypes of the *HLA-Cw6* and CDSN*TTC alleles were constructed in the 228 families by using Merlin and the biallelic genotypes (+/+, +/− and −/−) of the two loci. The recombinant haplotypes that carry either the *HLA-Cw6* or CDSN*TTC risk allele were identified and tested for association with psoriasis in the same 228 families. Subsequently, the recombination breakpoints of the recombinant haplotypes were mapped in the 163 families where 10 microsatellite markers (in addition to the *Cw6* and CDSN*TTC alleles) were genotyped. The recombination breakpoints were mapped to the last marker on both sides of the *CDSN* locus that bears a risk allele, to ensure that these recombinant haplotypes fully retained the portion of the candidate interval being tested for exclusion. TDT test was subsequently performed on the recombinant haplotypes sharing the same breakpoints to assess association between the different portions of the *PSORS1* candidate region and psoriasis. Due to the low frequency of the *HLA-Cw6* ^+^/CDSN*TTC ^−^ recombinant haplotypes, only the *HLA-Cw6* ^−^/CDSN*TTC ^+^ recombinant haplotypes were used in the analysis of identifying the maximum portion of the *HLA-Cw6* ^+^/CDSN*TTC ^+^ risk haplotype that is retained by the non-risk *HLA-Cw6* ^−^/CDSN*TTC ^+^ recombinant haplotypes.

### Combined Linkage and Association Analysis

To evaluate the contribution of the *HLA-Cw6* allele to the linkage evidence observed at the *PSORS1* locus, we performed the Linkage and Association Modeling in Pedigrees (LAMP) analysis [47] in the 163 families using 7 microsatellite markers surrounding the maximum LOD score peak identified in our previous genome-wide analysis as well as the *HLA-C* and *CDSN* loci. LAMP linkage test, direct association test and indirect association test have been performed to get the linkage signal and to assess whether there are other variants that can explain the linkage signal.

## Results

### Single-Locus Association Analysis

**Figure 1:**
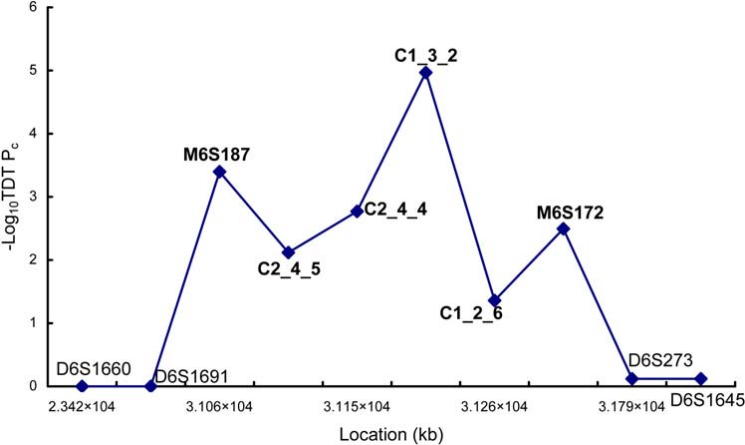
Single-Marker Association Analysis in 163 Families.

To follow up the linkage evidence at the *HLA* region (*PSORS1*) identified by our previous genome-wide analysis [37], we performed a family-based association analysis by genotyping 10 microsatellite markers from the *PSORS1* locus in 163 Chinese pedigrees (including the 61 families used in our genome-wide linkage analysis). As expected, the family-based TDT test revealed strong supporting evidence for our initial linkage finding (Fig 1). Of the 10 microsatellite markers analyzed, 5 markers (M6S187, C2_4_5, C2_4_4, C1_3_2 and M6S172) show strong evidence for association with psoriasis (TDT p<10^−3^), and one marker (C1_2_6) yields weak evidence for association (p = 0.0437). The results from the family-based TDT test are consistent with the results from the association analysis of the same 10 microsatellite markers in 192 unrelated cases and 192 controls (Supplementary Table 2), except at D6S273 locus where the association with psoriasis is suggested by the case-control analysis but not supported by the family-based analysis.

Association between the *HLA-Cw6* and CDSN*TTC ^+^ alleles and psoriasis was also investigated by TDT test in the same 163 families. The *HLA-Cw6* allele was determined by genotyping the seven coding SNPs that can uniquely define the *HLA-Cw6* allele, and the CDSN*TTC allele was determined by directly genotyping the three relevant SNPs (see Methods and Materials). The seven SNPs of the *HLA-C* locus yielded 9 haplotypes, and the three SNPs of the *CDSN* gene yielded 4 haplotypes. The haplotypes of each gene were tested for association with psoriasis by using TDT test, and the result is summarized in Table 2. As expected, only the haplotypes carrying either *HLA-Cw6* (*HLA-C* haplotype 11) or CDSN*TTC (*CDSN* haplotype 2) allele are positively associated with psoriasis. Both alleles are strongly associated with psoriasis, although *HLA-Cw6* allele seems to be more strongly associated with psoriasis than CDSN*TTC allele (85.6% vs. 73.9% transmission; 2.0×10^−19^ vs. 1.7×10^−8^ TDT p value) (Table 2).

**Table 2:** Association of Psoriasis with Alleles of HLA-C and CDSN.

Gene and Haplotype Number	Corresponding Allele(s)^a^	Haplotype Sequence^b^	Frequency^c^	T∶NT (%T)^d^	TDT^e^
HLA-C					
1	*01(0201–04, 06–13), *02(05), *08(12), *12(0301–07, 09, 11–13,15), *14(0202), and *16(01–0401, 08)	GCCTCCG	0.1142	12:34(26.1)	1.0×10^−3^
2	*01(05), *02(0201–04, 06–16), *03(14), *05(01–14), *07(07, 16), *08(0101–14), *12(0201–0203, 08, 14 and 16-18), *15(0201–17), *16(06–07), and *17(01–04)	GCCACCG	0.1399	35:45(43.8)	2.6×10^−1^
3	*03(0201–13, 16, and 18–29)	GCCACTG	0.1655	19:49(27.9)	3.0×10^−4^
4	*0315	GCAACTG	0.007	0:2(0)	4.9×10^−1^
5	*03(17), *14(0201, and 0203–07N, 08)	GCCTCTG	0.0536	3:22(12.0)	1.0×10^−4^
6	*04(010101–0104, 0401, 05, 08–10, 12–14, and 16–21)	GAAAGTG	0.0326	1:14(6.7)	1.0×10^−3^
11	*06(02–13)	CCATCCG	0.3893	137:23(85.6)	2.0×10^−19^
12	*07(0101–03, 05–06, 08–10, 13–15, and 17–30, 32N-39)	GCAACCG	0.0909	10:28(26.3)	3.0×10^−3^
13	*07(0401–0402 and 11–12)	CCAACCC	0.007	2:2(50.0)	1.0
CDSN					
1		TTT	0.1994	41:89(31.5)	2.0×10^−5^
2		TTC	0.4662	116:41(73.9)	1.7×10^−8^
4		TGC	0.1227	25:44(36.2)	2.2×10^−2^
6		CTC	0.2117	49:57(46.2)	4.4×10^−1^

a *HLA-C* allele designations follow the classification scheme of release 2.14.1 (Aug 2006) of the IMGT/HLA Sequence Database maintained by the HLA Informatics Group of the Anthony Nolan Research Institute. *CDSN* allele designations follow the classification scheme of Romphruk et al[49].

b Haplotype for seven coding SNPs of HLA-C (mRNA positions 213, 218, 341, 361, 387, 459, and 540) and three missense polymorphisms of CDSN (mRNA positions632, 1249, and 1256), in 5′→3′ orientation.

c Haplotype frequency, based on 889 founder chromosomes genotyped in 228 pedigrees for HLA-C and CDSN.

d For the multi-allelic TDT.

e All P values are uncorrected for multiple testing.

### Haplotype Association Analysis

We then performed a haplotype-based TDT analysis in the 163 families. The haplotypes were constructed using the genotypes of the 7 microsatellite markers showing single-locus association evidence as well as the *HLA-B, HLA-Cw6*, CDSN*TTC loci. After removing 31 inferred haplotypes, only 699 founder haplotypes from the 163 families were used in the following haplotype clustering and TDT test.

As described in Materials and Methods, the 699 founder haplotypes were grouped into 29 haplotype clusters where the last cluster (cluster 29) holds all the haplotypes that can not be clustered otherwise. All the haplotype clusters, except the cluster 29, were tested for association with psoriasis by using family-based TDT test, and the results are summarized in Table 3. The family-based association analysis of the haplotype clusters was also performed by using FBAT test, and the results are very consistent to the TDT results (data not shown). Of the 28 clusters tested, 4 haplotype clusters (cluster 4, 5, 6, and 7) are associated with risk. The four risk haplotypes share a 369-kb region of homologous marker alleles between HLA-B and M6S187 where the *HLA-Cw6* and CDSN*TTC risk alleles as well as the risk-associated alleles of the 5 microsatellite markers are all present on these four risk haplotypes. The four risk haplotypes seem to be derived from two known ancient risk haplotypes (*HLA-Cw6-B57* and *HLA-Cw6-B13*) [36], and the 369-kb interval is the minimum fragment of the ancestral haplotypes retained by all four risk haplotypes. The clusters 24 and 27 also show moderate evidence for protective effect. However, because the moderate evidence becomes insignificant after correction for multiple testing and the two clusters do not show any allele sharing, the evidence for the cluster 24 and 27 is likely to be false positive.

**Table 3:** 10-Marker Haplotype Clusters and Family-Based Test of Association with Psoriasis.

cluster^a^	Frequency^b^	Alleles										TDT	
		D6S273	HLA-B	HLA-C	M6S172	C1_2_6	C1_3_2	CDSN	C2_4_4	C2_4_5	M6S187	T∶NT(%T)^c^	P^d^
7	0.009	4	13	***2***	***1***	***1***	***2***	***TTC***	***2***	***3***	1	9:1(90.0)	1.1×10^−2^
4	0.065	2	57	***2***	***1***	***1***	***2***	***TTC***	***2***	***3***	1	42:7(85.7)	5.7×10^−7^
5	0.028	2	13	***2***	***1***	***1***	***2***	***TTC***	***2***	***3***	*3*	21:4(84.0)	7.0×10^−4^
6	0.018	3	13	***2***	***1***	***4***	***2***	***TTC***	***2***	***3***	3	9:2(81.8)	3.5×10^−2^
11	0.01	5	13	2	1	*1*	2	TTC	2	3	3	7:2(77.8)	9.6×10^−2^
25	0.016	2	15	1	1	1	*2*	TTC	2	3	*1*	10:3(76.9)	5.2×10^−2^
19	0.019	2	13	1	2	1	*4*	TTT	2	1	1	11:5(68.8)	1.3×10^−1^
3	0.013	1	13	2	1	1	2	TTC	2	3	*1*	8:4(66.7)	2.5×10^−1^
10	0.019	3	13	2	1	*6*	2	TTC	2	3	3	10:7(58.8)	4.7×10^−1^
20	0.015	*5*	13	1	2	1	4	TTT	2	1	*3*	10:7(58.8)	4.7×10^−1^
1	0.068	3	13	2	1	1	2	TTC	2	3	3	38:28(57.6)	2.2×10^−1^
2	0.034	3	13	2	1	1	2	TTC	2	3	2	23:17(57.5)	3.4×10^−1^
18	0.013	3	13	1	1	1	2	TTC	2	3	*3*	6:5(54.5)	7.6×10^−1^
8	0.018	3	13	2	1	5	2	TTC	2	3	*3*	10:10(50.0)	1
23	0.008	6	13	1	4	4	2	TGT	2	3	4	4:4(50.0)	1
14	0.019	*2*	15	1	4	4	2	TGT	2	3	*4*	11:13(45.8)	6.8×10^−1^
17	0.019	2	57	1	2	1	2	TTC	2	3	*5*	12:15(44.4)	5.6×10^−1^
16	0.009	5	58	1	3	1	3	TGT	*8*	3	2	6:8(42.9)	5.9×10^−1^
9	0.005	3	13	2	1	1	2	TTC	2	3	1	3:4(42.9)	7.1×10^−1^
28	0.008	3	27	1	2	*7*	4	TTT	2	1	2	3:4(42.9)	7.1×10^−1^
15	0.015	1	13	1	2	1	2	TTC	2	3	*5*	8:12(40.0)	3.7×10^−1^
13	0.017	4	15	1	2	1	2	TTC	2	3	*5*	6:11(35.3)	2.3×10^−1^
12	0.026	2	46	1	1	1	1	TTT	3	2	1	7:14(33.3)	1.3×10^−1^
26	0.007	2	35	1	1	*5*	6	TGT	4	3	*5*	3:6(33.3)	3.2×10^−1^
22	0.005	3	57	1	3	1	3	TGT	*8*	3	2	3:7(30.0)	2.1×10^−1^
21	0.009	6	44	1	1	*6*	4	TGC	6	2	4	2:7(22.2)	9.6×10^−2^
24	0.01	2	13	1	2	1	5	TGT	3	2	2	1:7(12.5)	3.4×10^−2^
27	0.004	2	67	1	4	4	2	TTC	2	3	1	0:4(0)	4.6×10^−2^

NOTE—The table displays all 9 markers subjected to haplotype clustering, along with HLA-B, in centromeric→telomeric order, from left to right. Italicized numbers indicate that the allele occurred in 50%–80% of the founder haplotypes comprising the cluster. Numbers in roman type indicate that the allele occurred in at least 80% of the founder haplotypes comprising the cluster. The alleles for HLA-B and CDSN are standard allele designations, whereas for HLA-C, 2 correspond to Cw6 alleles, and 1 corresponds to non-Cw6 alleles. The bold number area shows the minimum region of conserved by all risk haplotypes.

a All clusters among 699 founder chromosomes genotyped in 163 pedigrees are shown.

b Frequency of haplotypes in cluster.

c For the biallelic TDT.

d Uncorrected exact binomial P value for TDT.

Hence, the haplotype analysis determined a 369-kb critical region for the *PSORS1*. However, due to the strong linkage disequilibrium within this region, association evidences observed at the *HLA-Cw6* and CDSN*TTC alleles as well as other 5 marker alleles are not independent from each other, and the haplotype-based analysis can not discriminate which alleles is the primary cause of association with psoriasis observed within the *PSORS1* locus.

### Recombinant Haplotype Analysis

To evaluate which of the risk alleles within the 369-kb critical region is likely to be the primary cause of association, we employed the strategy of recombinant haplotype analysis where different risk allele or alleles are evaluated for association separately. First, we evaluated which of the *HLA-Cw6* or CDSN*TTC allele is more likely to be the primary *PSORS1* risk allele, by analyzing the recombinant haplotypes that have separated the *HLA-Cw6* and CDSN*TTC risk alleles in the 163 families as well as additional 65 affected sib pairs. After discarding 101 (10.2%) phase unknown haplotypes, the 889 founder haplotypes of the *HLA-Cw6* and CDSN*TTC alleles from the 228 pedigrees were analyzed by TDT test (Table 4). Of the 889 founder haplotypes, *HLA-Cw6* ^+^/CDSN*TTC ^+^ and *HLA-Cw6* ^−^/CDSN*TTC ^−^ haplotypes are both common with 32.6% and 52.6% frequency, respectively. As expected, the *HLA-Cw6* ^+^/CDSN*TTC ^+^ haplotype is strongly associated with risk for psoriasis (T∶NT = 129:34, p = 9.9×10^−14^), whereas the *HLA-Cw6* ^−^/CDSN*TTC ^−^ haplotype is clearly not associated (T∶NT = 50:117, p = 2.2×10^−7^). The *HLA-Cw6* ^−^/CDSN*TTC ^+^ recombinant haplotype is also common (12.9%) and is surely not associated with risk for psoriasis (T∶NT = 29:57, p = 0.0025). The *HLA-Cw6* ^+^/CDSN*TTC ^− ^recombinant haplotype is rare (1.8%) so that its association with risk can not be determined (Table 4). This result strongly indicates that the CDSN*TTC allele itself does not confer any risk without the presence of the *HLA-Cw6* allele and further suggests that the *PSORS1* gene is likely to be located within the *HLA-C* side of the 369 critical region.

**Table 4:** Association of Psoriasis with Recombination Haplotypes of HLA-Cw6 and CDSN*TTC Alleles.

haplotype	Frequency^a^	TDT	
		T∶NT (%T)^b^	P^c^
HLA-Cw6^+^/CDSN*TTC^+^	0.3262	129:34(79.1)	9.9×10^−14^
HLA-Cw6^+^/CDSN*TTC^−^	0.0180	5:4(55.6)	7.4×10^−1^
HLA-Cw6^−^/CDSN*TTC^+^	0.1294	29:57(33.7)	2.5×10^−3^
HLA-Cw6^−^/CDSN*TTC^−^	0.5264	50:117(29.9)	2.2×10^−7^

a Frequency of founder haplotypes are based on 889 chromosomes genotyped in 228 pedigrees.

b For the biallelic TDT.

c Uncorrected exact binomial P value for TDT.

We also performed a conditional association analysis using the WHAP program. A total of 90 trios were selected randomly from the 163 families. As shown in the table 5, only *HLA-Cw6* shows significant association evidence (empirical p-value from 1000 permutations = 9.9×10^−4^) after controlling for the genotypes of the surrounding loci (including the CDSN*TTC allele), whereas the CDSN*TTC allele no long shows significant association evidence after controlling for the genotypes of the surrounding loci (including the *HLA-Cw6* allele). Furthermore, the omnibus association test of the region becomes insignificant after controlling the effect of *HLA-Cw6* alleles (p = 0.14), whereas the same omnibus association test remains significant after controlling the effect of CDSN*TTC allele (p = 0.03). Being consistent with the results from the recombinant haplotype analysis, the conditional association analysis provides further supporting evidence that *HLA-C* is more likely than *CDSN* to be the candidate of the *PSORS1* gene or the *PSORS1* gene is more likely to be located nearby *HLA-C* than *CDSN* within the 369-kb critical region.

**Table 5:** Results of Conditional Association Analysis.

locus	Single marker analysis	Omnibus test excluding everything else	Omnibus test after controlling for locus
D6S273	1.9×10^−1^	0.79	–
HLA-C	1.9×10^−3^	9.9×10^−4^	0.14
M6S172	2.9×10^−2^	0.08	–
C1_2_6	3.1×10^−2^	0.36	–
C1_3_2	1.9×10^−3^	0.95	–
CDSN	3.9×10^−3^	0.23	0.03
C2_4_4	3.9×10^−3^	0.39	–
C2_4_5	7.4×10^−2^	0.08	–
M6S187	4.3×10^−1^	0.39	–

Empirical p-value from 1000 permutations were provided.

**Table 6:** 105^a^ Recombinant Haplotypes between the Risk Alleles of HLA-Cw6 and CDSN*TTC.

Haplotype Type	cluster	Alleles^c^								
		HLA-B	HLA-C	M6S172	C1_2_6	C1_3_2	CDSN	C2_4_4	C2_4_5	Number
Cw6+/CDSN*TTC+^b^	1	**13/57**	**2**	**1**	**1/4**	**2**	**TTC**	**2**	**3**	
										
Cw6+/CDSN*TTC-	1	**13**	**2**	**1**	**1**	**2**	-	-	-	1
	2	**13/57**	**2**	**1**	**1/4**	-	-	-	-	2
	3	-	**2**	**1**	-	-	-	-	-	2
	4	**13**	**2**	-	-	-	-	-	-	2
Cw6-/CDSN*TTC+	1	-	-	-	-	-	**TTC**	**2**	**3**	3
	2	-	-	-	-	**2**	**TTC**	**2**	**3**	7
	3	-	-	-	**1/4**	**2**	**TTC**	**2**	**3**	52
	4	-	-	**1**	**1/4**	**2**	**TTC**	**2**	**3**	29
	5	-	-	-	-	-	**TTC**	-	-	2
	6	-	-	-	-	**2**	**TTC**	**2**	-	1
	7	-	-	-	**4**	**2**	**TTC**	**2**	-	3
	8	-	-	**1**	**1**	**2**	**TTC**	**2**	-	1

a Of the 699 founder chromosomes from 163 pedigrees.

b Show all the risk alleles within the 369-kb critical region carried by all the Cw6+/CDSN*TTC+ risk haplotypes

c Bold alleles are the risk alleles within the 369-kb critical region of the Cw6+/CDSN*TTC+ risk haplotypes that are carried by the recombinant haplotypes; and ‘-’ indicates non-risk alleles.

To further refine the location of the *PSORS1* gene in Chinese population, we then performed an exclusion analysis within the 369-kb critical region (between *HLA-B* and M6S187), as demonstrated in Nair et al's study [14]. We first mapped the breakpoints of ancestral recombination between the *HLA-Cw6* and CDSN*TTC alleles in the 163 families. By analyzing the genotypes of 7 markers (*HLA-Cw6*, CDSN*TTC and 5 microsatellite markers) within the 369-kb region, 105 recombinant haplotypes between *HLA-Cw6* and CDSN*TTC loci were identified (among the 699 founder chromosomes) in the 163 families, including 7 *HLA-Cw6* ^+^/CDSN*TTC ^− ^haplotypes and 98 *HLA-Cw6* ^−^/CDSN*TTC ^+^ haplotypes (Table 6). Of the observed 105 recombinant haplotypes, 32 (30.5%) of the recombination breakpoints are mapped to the 48-kb region between *HLA-C* and M6S172, and 67 (63.8%) are mapped to the 92-kb region between M6S172 and C1_3_2. Only 6 (5.7%) recombination breakpoints are mapped to the 7-kb interval between C1_3_2 and *CDSN*. Hence, the recombination events between *HLA-C* and *CDSN* loci appear to be unevenly distributed and are largely limited to the 140-kb interval between *HLA-C* and C1_3_2 in the Chinese population. We then performed an exclusion analysis within the 369-kb critical region by identifying the maximum portion of the *HLA-Cw6* ^+^/CDSN*TTC ^+^ risk haplotype that is retained by the non-risk *HLA-Cw6* ^−^/CDSN*TTC ^+^ recombinant haplotypes. As shown in Table 7, the *HLA-Cw6* ^−^/CDSN*TTC ^+^ recombinant haplotypes that carry the C1_3_2-C2_4_5 portion of the *HLA-Cw6* ^+^/CDSN*TTC ^+^ risk haplotype are clearly not associated with risk to psoriasis (T∶NT = 28:47, p  = 0.028). It is also true for the *HLA-Cw6* ^−^/CDSN*TTC ^+^ recombinant haplotypes that carry the C1_2_6-C2_4_5 portion of the *HLA-Cw6* ^+^/CDSN*TTC ^+^ risk haplotype (T∶NT = 27:45, p  = 0.034). For the *HLA-Cw6* ^−^/CDSN*TTC ^+^ recombinant haplotypes that carry the M6S172-CDSN portion of the *HLA-Cw6* ^+^/CDSN*TTC ^+^ risk haplotype, our data suggested an under-transmission of the recombinant haplotypes (T∶NT = 11:20, p = 0.11), but the evidence is not statistically significant. Therefore, our results provided significant evidence for supporting the exclusion of the C1_2_6-C2_4_5 interval from the 369-kb critical region of the *PSORS1* locus. Our result also provides suggestive evidence to further exclude the M6172-C1_2_6 interval, but statistical evidence for this exclusion is moderate and needs to be confirmed by further study.

**Table 7:** Association Analysis of the HLA-Cw6^−^/CDSN*TTC ^+^ Recombinant Haplotypes Carrying Different Portions of the HLA-Cw6^+^/CDSN*TTC^+^ Risk Haplotype.

Test region^a^	number	Frequency^b^	TDT		
			T∶NT (%T)^c^	chi	p^d^
CDSN-C2_4_5	91	0.1302	28∶49(36.4)	5.7	1.7×10^−2^
C1_3_2-C2_4_5	88	0.1259	28∶47(37.3)	4.8	2.8×10^−2^
C1_2_6-C2_4_5	81	0.1159	27∶45(37.5)	4.5	3.4×10^−2^
M6S172-C2_4_5	29	0.0415	11∶20(35.5)	2.6	1.1×10^−1^

a To be conservative, the smallest possible portion of the extended risk haplotype carried by *HLA-Cw6* ^−^ /CDSN*TTC ^+^_recombinant haplotypes were used to determine whether a recombinant haplotype qualified for assessing the exclusion of the test region from the *PSORS1* candidate interval.

b Frequency of *HLA-Cw6* ^−^ /CDSN*TTC ^+^ recombinants carrying the test region; results are based on 699 founder chromosomes in 163 pedigrees.

c For the biallelic TDT.

d Uncorrected exact binomial P value for TDT.

### Combined Linkage and Association Analysis of the HLA-Cw6 Allele

Finally, we evaluated the contribution of the *HLA-Cw6* risk allele to the linkage evidence observed at the *PSORS1* locus. We performed a LAMP analysis in the 163 families by using the *HLA-Cw6* and CDSN*TTC alleles as well as the 7 microsatellite markers. As expected, the LAMP analysis revealed highly significant evidences for both linkage (LOD = 5.19, p = 2.6×10^−5^) and association (LOD = 45.88, p = 7.2×10^−48^), and both the evidences are mapped to the *HLA-Cw6* locus. After controlling for the effect of the *HLA-Cw6* allele, the linkage evidence is dramatically reduced, but still significant (LOD = 2.25, p = 0.005). This suggests that the *Cw6* allele is a major contributor to the linkage evidence observed at the *PSORS1* locus, but it cannot explain the full linkage evidence.

## Discussion

We have done the first fine mapping analysis of the *PSORS1* locus in Chinese population. Our single-locus association analysis provides strong supporting evidence for the *PSORS1* locus in a Chinese population and reveals a similar association pattern across the *PSORS1* region to that demonstrated in various populations. Our haplotype-based association analysis indicates that all the risk haplotypes within the *PSORS1* locus are *HLA-Cw6* positive and likely derived from two known ancient risk haplotypes (*HLA-Cw6-B57* and *HLA-Cw6-B13*) in the Chinese population, consistent with what has been found by previous studies in other populations. The *HLA-Cw7-B58* haplotype, which was found to be positively associated with psoriasis in a Sardinian population [10], was not present in the Chinese population. *B13-Cw1* and *B13-Cw3* haplotypes are present in our Chinese samples, but the sample size of these two haplotypes was too small to test for association with psoriasis. Allele comparison among all the risk haplotypes has identified a 369-kb critical region for *PSORS1* where all the risk-associated alleles of the 5 marker loci as well as *HLA-C* and *CDSN* loci are carried by the identified risk haplotypes, reflecting the extensive LD across the *PSORS1* region in the Chinese population as reported in other populations. The 369-kb critical region identified in Chinese population is very similar to the 300-kb critical region identified in a western population by similar methods [14].

Within the critical region, *HLA-C* and *CDSN* are the two leading candidates for the *PSORS1* gene both with strong supporting evidence. *HLA-Cw6* allele is the most significant marker for psoriasis risk prediction and has been indicated to be involved in the psoriasis-related immune response [19]. *CDSN* is the second extensively investigated candidate and codes corneodesmosin involved in keratinocyte cohesion and desquamation [48]. To evaluate which of the two genes is more likely to be the candidate, we performed the association analysis of the recombinant haplotypes that carry only one of the two risk alleles of the two genes. The frequencies of these two risk alleles in Chinese population are 39% and 47%, respectively, which are slightly higher than their frequencies in Caucasian population (21% and 23%, respectively) [14]. The frequency of the recombinant haplotypes of the *HLA-Cw6* and CDSN*TTC alleles is also higher in Chinese psoriatic families (14.7%) than in Caucasian psoriatic families (2.7%) [14]. The majority of the recombinant haplotypes are *HLA-Cw6* ^−^/CDSN*TTC ^+^ haplotypes (12.9%) that are clearly not associated with disease phenotype, suggesting that without the *HLA-Cw6* allele, CDSN*TTC does not confer any risk. This result is further supported by our conditional association analysis where only the *HLA-Cw6* allele shows independent effect from the genotypes of the surrounding marker loci including the CDSN*TTC allele. Therefore, our results have clearly excluded *CDSN* as the *PSORS1* susceptibility gene, which is consistent with the conclusion from Nair et al's study in Caucasian population [14].

We then attempted to further narrow down the location of the *PSORS1* gene within the 369-kb critical region by mapping the recombination breakpoints and performing an exclusion analysis, as illustrated in Nair et al's study [14]. The common region of the recombination breakpoints between *HLA-C* and *CDSN* loci in Chinese population (*HLA-C* to C1_3_2) is only partially overlapped with the one observed in Caucasian population (M6S169 to CDSN) where over 90% of recombination breakpoints were mapped [14]. In Caucasian population, most of the recombination breakpoints between *HLA-C* and *CDSN* loci are located within the region telomeric to C1_2_6 (M6S111) (89.5%), whereas in Chinese population, most of the recombination breakpoints are located within the region centromic to C1_2_6 (about 86.7%). Our results can exclude the C1_2_6-C2_4_5 interval from the 369-kb critical region of *PSORS1* with confidence. For the *HLA-Cw6* ^−^/CDSN*TTC ^+^ recombinant haplotypes that carry the M6S172-C2_4_5 portion of the *HLA-Cw6* ^+^/CDSN*TTC ^+^ risk haplotype, our result also indicates an under-transmission of the haplotypes (T∶NT = 11:20, p = 0.11), but the evidence is not statistically significant. Therefore, we can narrow down the *PSORS1* locus to the 172-kb region between *HLA-B* and C1_2_6 and potentially to the interval between *HLA-B* and M6S172, if the exclusion of the M6S172-C2_4_5 interval can be confirmed by further study in Chinese population. The more centromeric location of the recombination breakpoints in Chinese population (than in Caucasian population) allows us to narrow down the location of the *PSORS1* gene into a smaller region than Nair et al's study [14].

Within our conservatively mapped critical region of the *PSORS1* between *HLA-B* and C1_2_6, there are only two known genes *HLA-C* and *HCG27*. It has been confirmed by many studies, including our present study, that the *HLA-Cw6* allele is a strong susceptibility allele for psoriasis. But, there is limited evidence for *HCG27* to be the primary susceptibility gene for psoriasis. Direct sequence analysis of two risk haplotypes and eight non-risk haplotypes by Nair et al [14] in Caucasian population indicated that *HCG27* carried no alleles unique to the risk haplotypes. Besides these two known genes, there are a few ESTs as well as a few conserved non-coding sequences within the critical region. However, there is no supporting evidence for them to be functional as either coding or regulatory sequences. Searching in the UCSC Genome Browser (May 2006 version) also failed to identify any evidence of known microRNAs within this critical region. Therefore, our recombination mapping analysis has provided strong supporting evidence for *HLA-C* to be the primary susceptibility gene of the *PSORS1* locus.

*HLA-Cw6* is a major contributor to the linkage and association evidence observed at the *PSORS1* locus, but may not be the only risk allele within the *PSORS1* locus. The importance of the *HLA-Cw6* allele is demonstrated by the fact that after controlling for the effect of the *HLA-Cw6* allele, we could no longer identify significant association evidence at the *PSORS1* locus by the family-based test, and the supporting evidence for linkage to the *PSORS1* locus has also been dramatically reduced. However, the *HLA-Cw6* allele cannot fully explain all the linkage evidence, because the linkage evidence for the *PSORS1* locus is still significant after controlling for the effect of the *HLA-Cw6* allele. This result can be explained by two possibilities. The *HLA-Cw6* is not the only risk allele, and there are additional risk allele(s) within the region. Alternatively, the *HLA-Cw6* is only a marker allele and therefore indirectly associated with disease phenotype through LD with an unidentified causal allele. Although we can detect residual linkage evidence after controlling the effect of the *HLA-Cw6* allele, we could not identify independent association evidence from the *HLA-Cw6* allele in our family-based association study. This could be because our conditional family-based association analysis does not have sufficient power for detecting the residual association evidence due to the moderate number of informative families for family-based association test. It is very interesting to see that the *HLA-Cw6* can only partially explain the observed linkage evidence at the *PSORS1* locus, but the result needs to be confirmed by more studies.

**Figure 2:**
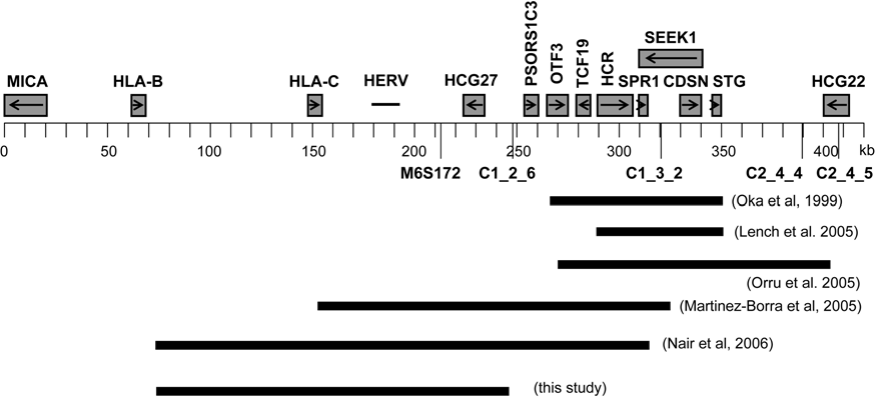
Comparison of the PSORS1 Candidate Regions Mapped by Different Studies. HERV: the fragments of a human endogenous retrovirus K (HERV-K) family.

Our fine mapping result is only partially consistent with the ones from previous studies. The minimal candidate region of *PSORS1* mapped by our study overlaps the regions mapped in an American Caucasian population [14] and a Jewish population [11], but is different from the ones mapped by other studies in Caucasian, Japanese and Sardinian psoriatic populations [9],[10],[12] (Fig 2). This difference could be due to the existence of different risk haplotypes in other populations. For example, The *HLA-Cw7-B58* recombinant was found to be positively associated with psoriasis in Sardinian samples but not present in our Chinese samples. The difference could also be due to the employment of different methodologies for fine mapping analysis. Another explanation is that there might be different susceptibility alleles in different populations. Giving the fact that psoriasis is a chronic inflammatory skin disorder and that many genes within the *PSORS1* region are known to be involved in autoimmune response and skin development, it is not implausible that there might be more than one gene within this region to play a role in the pathogenesis of psoriasis. It is therefore possible that different ethnic populations might carry different susceptibility alleles from different genes and that the results from the fine mapping studies done in different populations are not always consistent with each other due to the genetic heterogeneity of the *PSORS1* locus. Further studies will be needed to confirm this hypothesis.

## Supporting Information

Table S1 Clinical details of all pedigrees.
(0.04 MB DOC)

Table S2 Results of single-marker association study.
(0.05 MB DOC)

## References

[1] Nevitt GJ, Hutchinson PE (1996). Psoriasis in the community: prevalence, severity and patients' beliefs and attitudes towards the disease.. Br J Dermatol.

[2] Shao CG, Zhang GW, Wang GC (1987). Distribution of psoriasis in China: a nationwide screening.. Proc Chin Acad Med Sci Peking Union Med Coll.

[3] Nair RP, Henseler T, Jenisch S, Stuart P, Bichakjian CK (1997). Evidence for two psoriasis susceptibility loci (HLA and 17q) and two novel candidate regions (16q and 20p) by genome-wide scan.. Hum Mol Genet.

[4] Trembath RC, Clough RL, Rosbotham JL, Jones AB, Camp RD (1997). Identification of a major susceptibility locus on chromosome 6p and evidence for further disease loci revealed by a two stage genome-wide search in psoriasis.. Hum Mol Genet.

[5] Burden AD, Javed S, Bailey M, Hodgins M, Connor M (1998). Genetics of psoriasis: paternal inheritance and a locus on chromosome 6p.. J Invest Dermatol.

[6] Schmitt-Egenolf M, Eiermann TH, Boehncke WH, Stander M, Sterry W (1996). Familial juvenile onset psoriasis is associated with the human leukocyte antigen (HLA) class I side of the extended haplotype Cw6-B57-DRB1*0701-DQA1*0201-DQB1*0303: a population- and family-based study.. J Invest Dermatol.

[7] Jenisch S, Henseler T, Nair RP, Guo SW, Westphal E (1998). Linkage analysis of human leukocyte antigen (HLA) markers in familial psoriasis: strong disequilibrium effects provide evidence for a major determinant in the HLA-B/-C region.. Am J Hum Genet.

[8] Balendran N, Clough RL, Arguello JR, Barber R, Veal C (1999). Characterization of the major susceptibility region for psoriasis at chromosome 6p21.3.. J Invest Dermatol.

[9] Oka A, Tamiya G, Tomizawa M, Ota M, Katsuyama Y (1999). Association analysis using refined microsatellite markers localizes a susceptibility locus for psoriasis vulgaris within a 111 kb segment telomeric to the HLA-C gene.. Hum Mol Genet.

[10] Orru S, Giuressi E, Carcassi C, Casula M, Contu L (2005). Mapping of the major psoriasis-susceptibility locus (PSORS1) in a 70-Kb interval around the corneodesmosin gene (CDSN).. Am J Hum Genet.

[11] Martinez-Borra J, Brautbar C, Gonzalez S, Enk CD, Lopez-Vazquez A (2005). The region of 150 kb telometic to HLA-C is associated with psoriasis in the Jewish population.. J Invest Dermatol.

[12] Lench N, Iles MM, Mackay I, Patel R, Sagoo GS (2005). Single-point haplotype scores telomeric to human leukocyte antigen-C give a high susceptibility major histocompatability complex haplotype for psoriasis in a caucasian population.. J Invest Dermatol.

[13] Helms C, Saccone NL, Cao L, Daw JA, Cao K (2005). Localization of PSORS1 to a haplotype block harboring HLA-C and distinct from corneodesmosin and HCR.. Hum Genet.

[14] Nair RP, Stuart PE, Nistor I, Hiremagalore R, Chia NV (2006). Sequence and haplotype analysis supports HLA-C as the psoriasis susceptibility 1 gene.. Am J Hum Genet.

[15] Mallon E, Newson R, Bunker CB (1999). HLA-Cw6 and the genetic predisposition to psoriasis: a meta-analysis of published serologic studies.. J Invest Dermatol.

[16] Allen MH, Veal C, Faassen A, Powis SH, Vaughan RW (1999). A non-HLA gene within the MHC in psoriasis.. Lancet.

[17] Asumalahti K, Laitinen T, Itkonen-Vatjus R, Lokki ML, Suomela S (2000). A candidate gene for psoriasis near HLA-C, HCR (Pg8), is highly polymorphic with a disease-associated susceptibility allele.. Hum Mol Genet.

[18] Nair RP, Stuart P, Henseler T, Jenisch S, Chia NV (2000). Localization of psoriasis-susceptibility locus PSORS1 to a 60-kb interval telomeric to HLA-C.. Am J Hum Genet.

[19] Carlen L, Sakuraba K, Stahle M, Sanchez F (2007). HLA-C expression pattern is spatially different between psoriasis and eczema skin lesions.. J Invest Dermatol.

[20] Burden AD (2000). Identifying a gene for psoriasis on chromosome 6 (PSORS1).. Br J Dermatol.

[21] Allen M, Ishida-Yamamoto A, McGrath J, Davison S, Iizuka H (2001). Corneodesmosin expression in psoriasis vulgaris differs from normal skin and other inflammatory skin disorders.. Lab Invest.

[22] Ameen M, Allen MH, Fisher SA, Lewis CM, Cuthbert A (2005). Corneodesmosin (CDSN) gene association with psoriasis vulgaris in Caucasian but not in Japanese populations.. Clin Exp Dermatol.

[23] Capon F, Allen MH, Ameen M, Burden AD, Tillman D (2004). A synonymous SNP of the corneodesmosin gene leads to increased mRNA stability and demonstrates association with psoriasis across diverse ethnic groups.. Hum Mol Genet.

[24] Asumalahti K, Veal C, Laitinen T, Suomela S, Allen M (2002). Coding haplotype analysis supports HCR as the putative susceptibility gene for psoriasis at the MHC PSORS1 locus.. Hum Mol Genet.

[25] O'Brien KP, Holm SJ, Nilsson S, Carlen L, Rosenmuller T (2001). The HCR gene on 6p21 is unlikely to be a psoriasis susceptibility gene.. J Invest Dermatol.

[26] Tiala I, Suomela S, Huuhtanen J, Wakkinen J, Holtta-Vuori M (2007). The CCHCR1 (HCR) gene is relevant for skin steroidogenesis and downregulated in cultured psoriatic keratinocytes.. J Mol Med.

[27] Elomaa O, Majuri I, Suomela S, Asumalahti K, Jiao H (2004). Transgenic mouse models support HCR as an effector gene in the PSORS1 locus.. Hum Mol Genet.

[28] Suomela S, Elomaa O, Asumalahti K, Kariniemi AL, Karvonen SL (2003). HCR, a candidate gene for psoriasis, is expressed differently in psoriasis and other hyperproliferative skin disorders and is downregulated by interferon-gamma in keratinocytes.. J Invest Dermatol.

[29] Chang YT, Chou CT, Shiao YM, Lin MW, Yu CW (2006). Psoriasis vulgaris in Chinese individuals is associated with PSORS1C3 and CDSN genes.. Br J Dermatol.

[30] Holm SJ, Sanchez F, Carlen LM, Mallbris L, Stahle M (2005). HLA-Cw*0602 associates more strongly to psoriasis in the Swedish population than variants of the novel 6p21.3 gene PSORS1C3.. Acta Derm Venereol.

[31] Holm SJ, Carlen LM, Mallbris L, Stahle-Backdahl M, O'Brien KP (2003). Polymorphisms in the SEEK1 and SPR1 genes on 6p21.3 associate with psoriasis in the Swedish population.. Exp Dermatol.

[32] Veal CD, Capon F, Allen MH, Heath EK, Evans JC (2002). Family-based analysis using a dense single-nucleotide polymorphism-based map defines genetic variation at PSORS1, the major psoriasis-susceptibility locus.. Am J Hum Genet.

[33] Foerster J, Nolte I, Junge J, Bruinenberg M, Schweiger S (2005). Haplotype sharing analysis identifies a retroviral dUTPase as candidate susceptibility gene for psoriasis.. J Invest Dermatol.

[34] Walsh EC, Mather KA, Schaffner SF, Farwell L, Daly MJ (2003). An integrated haplotype map of the human major histocompatibility complex.. Am J Hum Genet.

[35] Gonzalez S, Martinez-Borra J, Del Rio JS, Santos-Juanes J, Lopez-Vazquez A (2000). The OTF3 gene polymorphism confers susceptibility to psoriasis independent of the association of HLA-Cw*0602.. J Invest Dermatol.

[36] Jenisch S, Koch S, Henseler T, Nair RP, Elder JT (1999). Corneodesmosin gene polymorphism demonstrates strong linkage disequilibrium with HLA and association with psoriasis vulgaris.. Tissue Antigens.

[37] Zhang XJ, He PP, Wang ZX, Zhang J, Li YB (2002). Evidence for a major psoriasis susceptibility locus at 6p21(PSORS1) and a novel candidate region at 4q31 by genome-wide scan in Chinese hans.. J Invest Dermatol.

[38] Zhang XJ, Zhang AP, Yang S, Gao M, Wei SC (2003). Association of HLA class I alleles with psoriasis vulgaris in southeastern Chinese Hans.. J Dermatol Sci.

[39] Bunce M, Barnardo MC, Procter J, Marsh SG, Vilches C (1997). High resolution HLA-C typing by PCR-SSP: identification of allelic frequencies and linkage disequilibria in 604 unrelated random UK Caucasoids and a comparison with serology.. Tissue Antigens.

[40] Miller SA, Dykes DD, Polesky HF (1988). A simple salting out procedure for extracting DNA from human nucleated cells.. Nucleic Acids Res.

[41] Chen JJ, Huang W, Gui JP, Yang S, Zhou FS (2005). A novel linkage to generalized vitiligo on 4q13-q21 identified in a genomewide linkage analysis of Chinese families.. Am J Hum Genet.

[42] Wigginton JE, Abecasis GR (2005). PEDSTATS: descriptive statistics, graphics and quality assessment for gene mapping data.. Bioinformatics.

[43] Abecasis GR, Cherny SS, Cookson WO, Cardon LR (2002). Merlin–rapid analysis of dense genetic maps using sparse gene flow trees.. Nat Genet.

[44] Benjamini Y, Drai D, Elmer G, Kafkafi N, Golani I (2001). Controlling the false discovery rate in behavior genetics research.. Behav Brain Res.

[45] Curtis D (1997). Use of siblings as controls in case-control association studies.. Ann Hum Genet.

[46] Purcell S, Daly MJ, Sham PC (2007). WHAP: haplotype-based association analysis.. Bioinformatics.

[47] Li M, Boehnke M, Abecasis GR (2005). Joint modeling of linkage and association: identifying SNPs responsible for a linkage signal.. Am J Hum Genet.

[48] Guerrin M, Simon M, Montezin M, Haftek M, Vincent C (1998). Expression cloning of human corneodesmosin proves its identity with the product of the S gene and allows improved characterization of its processing during keratinocyte differentiation.. J Biol Chem.

