# Supplementary material for: Fine Mapping of the Psoriasis Susceptibility Locus PSORS1 Supports HLA-C as the Susceptibility Gene in the Han Chinese Population
Source: PLoS Genet. 2008 Mar 21;4(3):e1000038. doi: 10.1371/journal.pgen.1000038 (PMC2265413; doi:10.1371/journal.pgen.1000038)
Supplement: Table S1 — Clinical details of all pedigrees. (0.04 MB DOC) [file pgen.1000038.s001.doc]

**Supplementary Table 1**. Clinical details of all pedigrees

| Clinical characteristics | Number. of individuals | | total |
| --- | --- | --- | --- |
|  | affected | unaffected |  |
| **Gender** | 549 | 444 | 993 |
| male | 321 | 198 |  |
| female | 228 | 246 |  |
| **Onset** |  |  |  |
| Mean age of onset (years) | 22.9y (6 months to 70 years) |  |  |
| **Average number per family** | 2.4 |  |  |
| **Mean numbers of affected sites** | 4.09 ± 2.12 |  |  |
| **PASI score** |  |  |  |
| ND | 56 |  |  |
| Mild (0–3) | 109 |  |  |
| Moderate (>3–15) | 233 |  |  |
| Severe (>15) | 151 |  |  |
| **Phenotype** |  |  |  |
| guttate | 82 |  |  |
| plaques | 467 |  |  |

The severity of the psoriasis was assessed by the PASI score, a PASI score below 3 was defined as mild, between 3 and 15 as moderate, and above 15 as severe disease.
