# Supplementary material for: Fine Mapping of the Psoriasis Susceptibility Locus PSORS1 Supports HLA-C as the Susceptibility Gene in the Han Chinese Population
Source: PLoS Genet. 2008 Mar 21;4(3):e1000038. doi: 10.1371/journal.pgen.1000038 (PMC2265413; doi:10.1371/journal.pgen.1000038)
Supplement: Table S2 — Results of single-marker association study. (0.05 MB DOC) [file pgen.1000038.s002.doc]

**Supplementary Table 2**. Results of single-marker association analysis.

|  |  |  | Frequency | |  | P-value | | |  |
| --- | --- | --- | --- | --- | --- | --- | --- | --- | --- |
| loci | location（NCBI Build 36） | Allele | cases | controls |  | P | FDR^b^ (BH) thresholds | OR | 95%CI of OR |
| D6S1660 | 23421875-23422085 | 3 | 151 | 137 |  | 1.3 × 10^-1^ | 4.2 × 10^-2^ | 1.25 | 0.93-1.68 |
| D6S1691 | 24032524-24032754 | 10 | 34 | 47 |  | 2.5 × 10^-1^ | 4.4 × 10^-2^ | 0.77 | 0.49-1.21 |
| **M6S187^a^** | 31060684-31060845 | 1 | 140 | 108 |  | 2.1 × 10^-2^ | **3.9 × 10^-2^** | 1.43 | 1.06-1.94 |
|  |  | 2 | 86 | 116 |  | 1.4 × 10^-2^ | **3.6 × 10^-2^** | 0.67 | 0.48-0.92 |
|  |  | 3 | 107 | 64 |  | 1.9 × 10^-4^ | **2.2 × 10^-2^** | 1.93 | 1.36-2.74 |
| **C2_4_5^a^** | [31130688- 31131123](http://www.ensembl.org/Homo_sapiens/contigview?&chr=6&vc_start=31120688&vc_end=31141123) | 3 | 259 | 191 |  | 6.3 × 10^-7^ | **1.1 × 10^-2^** | 2.09 | 1.56-2.81 |
| **C2_4_4^a^** | [31145709- 31145939](http://www.ensembl.org/Homo_sapiens/contigview?&chr=6&vc_start=31135709&vc_end=31155939) | 2 | 270 | 211 |  | 1.1 × 10^-5^ | **1.4 × 10^-2^** | 1.94 | 1.44-2.61 |
| **C1_3_2^a^** | 31190849-31196202 | 2 | 220 | 114 |  | 1.2 × 10^-14^ | **2.8 × 10^-3^** | 3.18 | 2.36-4.28 |
|  |  | 3 | 20 | 49 |  | 2.5 × 10^-4^ | **2.5 × 10^-2^** | 0.38 | 0.22-0.65 |
| **C1_2_6^a^** | 31257346- 31257644 | 1 | 228 | 193 |  | 1.1 × 10^-2^ | **3.3 × 10^-2^** | 1.45 | 1.09-1.92 |
|  |  | 4 | 83 | 33 |  | 4.7 × 10^-7^ | **8.3 × 10^-3^** | 2.93 | 1.91-4.52 |
| **M6S172^a^** | 31296057-31296231 | 1 | 110 | 60 |  | 1.4 × 10^-5^ | **1.7 × 10^-2^** | 2.17 | 1.52-3.09 |
|  |  | 8 | 11 | 37 |  | 1.1 × 10^-4^ | **1.9 × 10^-2^** | 0.28 | 0.14-0.55 |
| **D6S273^a^** | 31791664-31791799 | 2 | 123 | 56 |  | 1.1 × 10^-8^ | **5.6 × 10^-3^** | 2.76 | 1.94-3.94 |
|  |  | 3 | 32 | 61 |  | 1.0 × 10^-3^ | **2.8 × 10^-2^** | 0.48 | 0.31-0.76 |
|  |  | 4 | 137 | 140 |  | 8.2 × 10^-1^ | 4.7 × 10^-2^ | 0.97 | 0.72-1.29 |
| D6S1645 | 35690784-35691025 | 1 | 67 | 66 |  | 9.2 × 10^-1^ | 5.0 × 10^-2^ | 1.02 | 0.70-1.48 |

a. the markers which have risk allele associated with psoriasis.

b. FDR thresholds for which the corresponding nominal p-values remain significant after corrected for multiple tests are indicated in bold, 0.05 level used for FDR statistical significance.
